# Letter to the Editor: Delayed Presentation of Non-COVID-19 Patients During the COVID-19 Pandemic Is Not Limited to Children

**DOI:** 10.5041/RMMJ.10447

**Published:** 2021-07-20

**Authors:** Klaus Rose, Oishi Tanjinatus, Jane Grant-Kels, Earl B. Ettienne, Pasquale Striano

**Affiliations:** 1 klausrose Consulting, Riehen, Switzerland; 2 Howard University College of Pharmacy, Washington, DC, USA; 3 Dermatology Department, UConn Health, Farmington, CT, USA; 4 Pediatric Neurology and Muscular Diseases Unit, Department of Neurosciences, Rehabilitation, Ophthalmology, Genetics, Maternal and Child Health, University of Genoa, ‘G. Gaslini’ Institute, Genova, Italy

**Keywords:** COVID-19 pandemic, multiple inflammatory syndrome (MIS), multiple inflammatory syndrome in children (MIS-C), pediatric drug development

## TO THE EDITOR

We read with interest the report about four minors who were diagnosed late with non-COVID-19 diseases during the COVID-19 pandemic.^1^ We would like to emphasize that, firstly, such delays are not limited to minors, and secondly, that also in minors should we distinguish the administrative and the physiological meanings of the term “child” and hence distinguish administratively defined “children” who bodily are already mature from those young patients who bodily are indeed still children. The 16-year-old patient that was presented to the emergency room with endocarditis was bodily no longer a child, although administratively and probably also psychologically, due to his Down syndrome, he was still a child.^1^ Two of the other patients, one with hemolytic anemia (2.5 years old) and one with Ewing sarcoma (4 years old), were still pre-pubertal children, while the 13-year-old minor with a septic hip was already adolescent.^1^ The author of the cited paper works in a pediatric department and reports those patients that he has seen during his work. However, in our view there is nothing specifically pediatric in his observations. Several recent papers discuss delays of diagnosis and treatment of non-COVID-19 diseases during the pandemic, including head and neck cancer,^2^ appendicitis,^3^,^4^ heart failure and septicemia,^5^ pulmonary thromboembolism,^6^ pyelonephritis,^7^ and cancer in general.^8^ Some patients in these papers are administratively still “children,”^1^,^3^,^7^ some are adults, and appendicitis is discussed in both.^3^,^4^ The delay the COVID-19 pandemic has caused in the timely diagnosis of various diseases is not a “pediatric” challenge, but a challenge for medicine in general.

There is an exaggerated focus on “pediatric” COVID-19 long-term sequelae such as multiple inflammatory syndrome (MIS) in “children” (“MIS-C”), as opposed to adults (“MIS-A”), based first on observations by pediatricians and then on a general warning by the Centers of Diseases Control and Prevention (CDC). Multiple inflammatory syndrome (MIS) occurs in adult and minor patients of any age. The terms “MIS-C” and “MIS-A” should be abandoned and be replaced by the correct term, “MIS.”^9^ The above-mentioned pyelonephritis occurred in a 16-year-old adolescent and was first misdiagnosed as “MIS-C,”^7^ which further shows the potential dangers of the flawed CDC warning.^9^ That a pediatrician observes delays in diagnosis neither makes all observed patients “children,” nor does it make their diseases “pediatric.”^9^,^10^ Modern medicine has still to learn to distinguish between the classification of minors for administrative purposes, which is necessary in any modern complex differentiated society, and the flawed classification of legally defined minors as “children,” which leads to inclusion of minors into questionable “pediatric” studies that often harm.^10^

One further consequence of the blur between the administrative and the physiological classification of adolescents as “children” is that current COVID-19 vaccines are limited to ≥16 or ≥18-year-olds. We could discuss without end whose fault was the non-inclusion of adolescents into the pivotal vaccination studies: the developing companies, or the regulatory authorities?^9^,^10^ Who was first, the hen or the egg? Instead, on a pragmatic level, regulatory authorities worldwide, including Israel, should consider expanding the age range of vaccinations to adolescents ≥10 or ≥12 years old now, and to invite the producers to smart, short studies in younger minors to confirm the doses calculated on the basis of physiology-based pharmacokinetic (PBPK) modeling.

Children in modern societies reflect the retreat from parents’ unlimited jurisdiction towards perceiving their welfare as a shared social responsibility, resulting in societal investments in education, health care, and more. Children’s development is influenced both by their family and by society’s forces and norms.^11^ Adolescence, the phase between childhood and adulthood, encompasses elements of biological growth and major social role transitions, both of which have changed in the recent past. Puberty now begins earlier, while understanding of continued growth has lifted its endpoint age well into the 20s.^12^,^13^ When we use the term “child,” we need to be more aware than ever of its different administrative and physiological meanings.^10^
